# Predicting the potential distribution of *Dendrolimus punctatus* and its host *Pinus massoniana* in China under climate change conditions

**DOI:** 10.3389/fpls.2024.1362020

**Published:** 2024-05-24

**Authors:** Yijie Wang, Youjie Zhao, Guangting Miao, Xiaotao Zhou, Chunjiang Yu, Yong Cao

**Affiliations:** College of Big Data and Intelligent Engineering, Southwest Forestry University, Kunming, China

**Keywords:** climate change, *Dendrolimus punctatus*, *Pinus massoniana*, species distribution modeling, MaxEnt model

## Abstract

**Introduction:**

*Dendrolimus punctatus*, a major pest endemic to the native *Pinus massoniana* forests in China, displays major outbreak characteristics and causes severe destructiveness. In the context of global climate change, this study aims to investigate the effects of climatic variations on the distribution of *D. punctatus* and its host, *P. massoniana*.

**Methods:**

We predict their potential suitable distribution areas in the future, thereby offering a theoretical basis for monitoring and controlling *D. punctatus*, as well as conserving *P. massoniana* forest resources. By utilizing existing distribution data on *D. punctatus* and *P. massoniana*, coupled with relevant climatic variables, this study employs an optimized maximum entropy (MaxEnt) model for predictions. With feature combinations set as linear and product (LP) and the regularization multiplier at 0.1, the model strikes an optimal balance between complexity and accuracy.

**Results:**

The results indicate that the primary climatic factors influencing the distribution of *D. punctatus* and *P. massoniana* include the minimum temperature of the coldest month, annual temperature range, and annual precipitation. Under the influence of climate change, the distribution areas of *P. massoniana* and its pests exhibit a high degree of similarity, primarily concentrated in the region south of the Qinling−Huaihe line in China. In various climate scenarios, the suitable habitat areas for these two species may expand to varying degrees, exhibiting a tendency to shift toward higher latitude regions. Particularly under the high emission scenario (SSP5-8.5), *D. punctatus* is projected to expand northwards at the fastest rate.

**Discussion:**

By 2050, its migration direction is expected to closely align with that of *P. massoniana*, indicating that the pine forests will continue to be affected by the pest. These findings provide crucial empirical references for region-specific prevention of *D. punctatus* infestations and for the rational utilization and management of *P. massoniana* resources.

## 1 Introduction

The *Dendrolimus punctatus*, a member of the *Lasiocampidae* family within the *Lepidoptera* order, is a widespread coniferous tree leaf-eating pest in the forest ecosystems of southern China. It affects an area of over one million acres and is one of the most broadly distributed and severely damaging pests in the country (Chen, 2013; Li et al., 2019; Zhang et al., 2020). As a longstanding pest in Chinese forest ecosystems, its primary target is *Pinus massoniana* (Chai, 1995; Zhang et al., 2017; Niu and Fan, 2023), *P. massoniana*, a native Chinese tree species, is distinguished by its robust adaptability, rapid growth rate, and drought resistance. It stands as one of the primary species used for afforestation on barren hills and plays a crucial role in ecological construction projects (Liu et al., 2015; Yang et al., 2016; Meng et al., 2018). However, during outbreaks, *D. punctatus* can rapidly devastate pine forests, leading to widespread death of *P. massoniana* within a matter of days. These outbreaks cause ecological imbalances and substantial economic losses. Consequently, they pose a severe threat to the safety of forest ecosystems and the sustainability of forestry.

Global climate change, particularly alterations in temperature and precipitation, is a pivotal factor affecting the geographical distribution of species (Guo et al., 2014). The Intergovernmental Panel on Climate Change (IPCC)’s Sixth Assessment Report indicates that the global surface temperature from 2011 to 2020 was 1.1°C higher than the average from 1850 to 1900, with projections of a continual rise in temperature over the coming decades (Calvin et al., 2023). This warming trend is anticipated to increase precipitation levels and lead to alterations in river basins and adjustments in forest community structures (Shu et al., 2022; Chen et al., 2023; Zhao et al., 2024). As a poikilothermic organism, *D. punctatus* is highly sensitive to changes in environmental temperature and precipitation. These factors are decisive in the distribution and outbreak patterns of *D. punctatus*; temperature influences its developmental rate, and precipitation contributes to the survival of eggs and larvae (Lian et al., 2022). Future climate changes may exacerbate the spread of forest pests and significantly impact plant growth (Tang et al., 2021; Johnson and Haynes, 2023). Therefore, in the context of global climate change, studying the adaptability of *D. punctatus* and *P. massoniana* to future climatic conditions and their distributional changes is crucial for developing effective control strategies, protecting forest resources, and maintaining ecological balance.

Species distribution models are mathematical models based on species presence or abundance data, as well as environmental factors, utilized to assess and predict the potential impact of climate change on species (Guisan and Zimmermann, 2000). Common species distribution models include bioclimatic modeling (BIOCLIM), the genetic algorithm for rule-set prediction, generalized linear models, random forests, and the maximum entropy (MaxEnt) model (Phillips et al., 2006; Elith and Leathwick, 2009; Belgiu and Drăguţ, 2016; Yang et al., 2020). The MaxEnt model is considered one of the best-performing non-ensemble methods in niche modeling. Compared with other models, MaxEnt offers several advantages, such as the ability to utilize continuous and categorical data, and accounts for interactions among different variables. Moreover, MaxEnt can provide high accuracy even in scenarios where distribution data are scarce or incomplete (Elith and Leathwick, 2009; Warren and Seifert, 2011; Elith et al., 2015). Consequently, this model has extensive applications in various fields such as biodiversity conservation, the assessment of risks associated with invasive species, endangered species protection, impact assessments of climate change, and the prediction of quarantine pests (Cao et al., 2020; Li et al., 2020; Wan et al., 2020; Shi et al., 2023).

In this study, we used the MaxEnt model to simulate the impact of climate change on the suitable distribution of *D. punctatus* and *P. massoniana*, aiming to provide a scientific basis for the protection of *P. massoniana* forest resources and effective control of *D. punctatus*. The specific objectives of the study include: (1) predicting the potential distribution areas of these two species in China; (2) analyzing the impact of major environmental factors on their distribution; (3) forecasting and comparing the suitable habitats and trends of change in 2050 and 2070 under different climate scenarios; and (4) systematically analyzing the spatiotemporal changes in their distribution centroids caused by climate change.

## 2 Data sources and preprocessing

### 2.1 Species occurrence data

Data for *D. punctatus* and *P. massoniana* were obtained from the Center for Agriculture and Bioscience International (CABI: https://www.cabi.org), the Global Biodiversity Information Facility (GBIF: https://www.gbif.org/), the Chinese Virtual Herbarium (CVH: https://www.cvh.ac.cn/), and relevant published literature.

**Figure 1:**
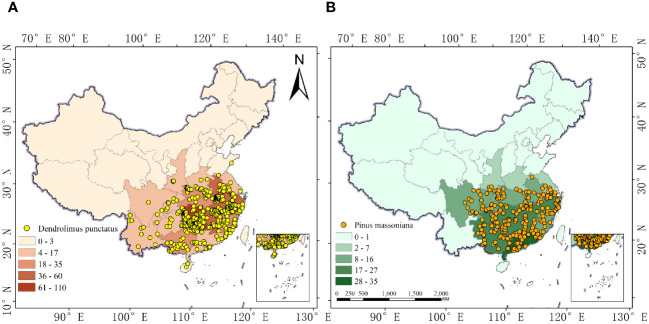
Occurrence records of *D. punctatus* and *P. massoniana* in China. **(A)** *D. punctatus*; **(B)** *P. massoniana*.

To avoid autocorrelation among samples, we further processed the distribution information of the target species. Initially, the Baidu Maps coordinate picker system was used to acquire precise longitudes and latitudes for collection points missing this information. The distribution point data in degrees, minutes, and seconds format were then converted into decimal (floating-point) numbers. Finally, to reduce spatial autocorrelation between sample points and decrease the errors in the model’s outcomes, we established a 5 km × 5 km grid (corresponding to the 2.5 arc-minute environmental data detailed in Section 2.2), retaining only one sampling point per grid and eliminating duplicate and sea-based points. The final number of sample points used for building the species distribution model was 429 for *D. punctatus* and 236 for *P. massoniana* (**Figure 1**).

### 2.2 Environmental data

**Figure 2:**
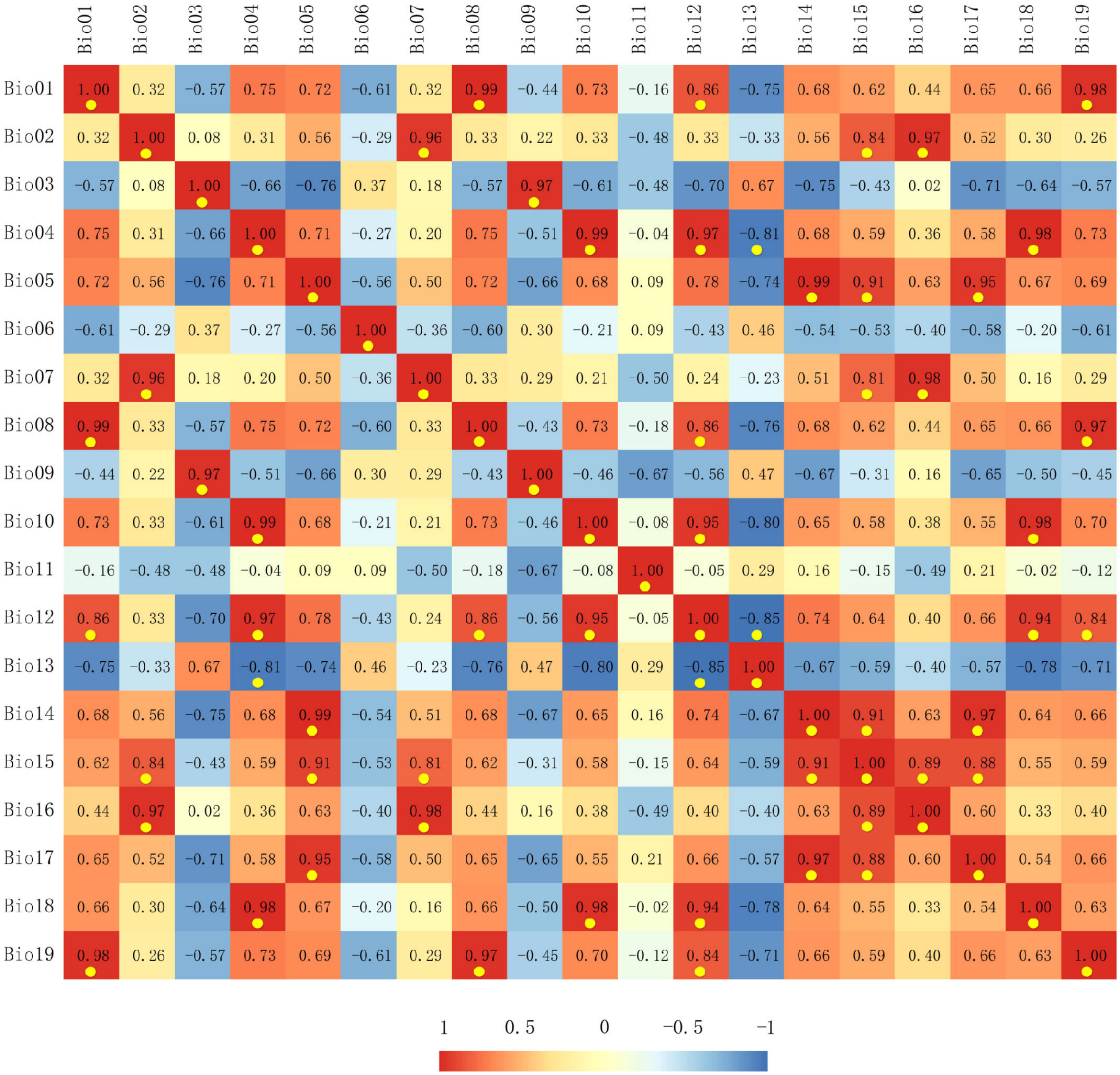
Correlation analysis results of the environmental variables. Red indicates a positive correlation, blue indicates a negative correlation, and highly correlated environmental variables (|r| > 0.8) are highlighted with yellow dots.

We selected 19 bioclimatic variables (BIO01-BIO19, **Table 1**) from the WorldClim database (version 2.1, spanning 1970–2000) (http://www.worldclim.org/) as the current climate data, with a spatial resolution of 2.5 arc minutes. To reduce multicollinearity among variables and prevent model overfitting, it was necessary to filter these 19 environmental variables (Morales et al., 2017). Initially, we imported the distribution data of the target species and the 19 environmental factors into MaxEnt, utilizing default parameters for pre-training, and a jackknife method to determine the contribution rate of each environmental factor (**Table 1**). We subsequently conducted a correlation analysis of the 19 environmental factors using ArcGIS (Esri) (**Figure 2**). If two or more environmental variables exhibited a high correlation (|r| > 0.8), we retained the variable with a higher contribution to the model and excluded those with a contribution rate of zero. Ultimately, six climatic variables were selected for modeling the potential distribution of the pine caterpillar and pine, namely, isothermality (BIO03), the minimum temperature of the coldest month (BIO06), the annual temperature range (BIO07), the mean temperature of the coldest quarter (BIO11), annual precipitation (BIO12), and the precipitation of driest month (BIO14).

**Table 1:** Description of climate variables and their pre-training contributions used for simulating the potential distribution of *Dendrolimus punctatus* and *Pinus massoniana*.

Variable	Climate variable	Contribution of *D. punctatus* (%)	Contribution of *P. massoniana* (%)
bio01	Annual mean temperature	1.9	0.6
bio02	Mean diurnal range	1.1	1.1
**bio03**	**Isothermality**	**0.6**	**3.6**
bio04	Temperature seasonality	5.5	3.0
bio05	Maximum temperature of the warmest month	4.6	0.8
**bio06**	**Minimum temperature of the coldest month**	**1.2**	**1.7**
**bio07**	**Annual temperature range**	**2.6**	**1.3**
bio08	Mean temperature of the wettest quarter	0.6	0.5
bio09	Mean temperature of the driest quarter	0.2	0.3
bio10	Mean temperature of the warmest quarter	1.6	1.5
**bio11**	**Mean temperature of the coldest quarter**	**1**	**0.4**
**bio12**	**Annual precipitation**	**4.2**	**10.8**
bio13	Precipitation of the wettest month	0.5	0.2
**bio14**	**Precipitation of the driest month**	**70**	**72.2**
bio15	Precipitation seasonality	1.8	0.6
bio16	Precipitation of the wettest quarter	0.3	0.2
bio17	Precipitation of the driest quarter	0.5	0.4
Bio18	Precipitation of the warmest quarter	0.2	0.1
Bio19	Precipitation of the coldest quarter	1.5	0.7

Selected climate variables are highlighted in bold and gray.

Three global circulation models (GCMs) from the Sixth Coupled Model Intercomparison Project (CMIP6), namely, BCC-CSM2-MR (Beijing Climate Center Climate System Model), MIROC6 (Model for Interdisciplinary Research on Climate), and CMCC-ESM2 (Centro Euro-Mediterraneo sui Cambiamenti Climatici Earth System Model 2) were selected as the future climate models. To mitigate the uncertainty associated with reliance on a single GCM, we averaged the occurrence probabilities of these three models. Based on this, we employed the Shared Socioeconomic Pathways (SSPs) SSP1-2.6 and SSP5-8.5, representing sustainable and business-as-usual development scenarios (Riahi et al., 2017; Liu et al., 2021), respectively, applied to the years 2041–2060 (2050s) and 2061–2080 (2070s). These choices of models and SSPs aimed to explore the potential distribution changes of the pine caterpillar and pine under different future development pathways.

## 3 Research methods

### 3.1 Research framework

**Figure 3:**
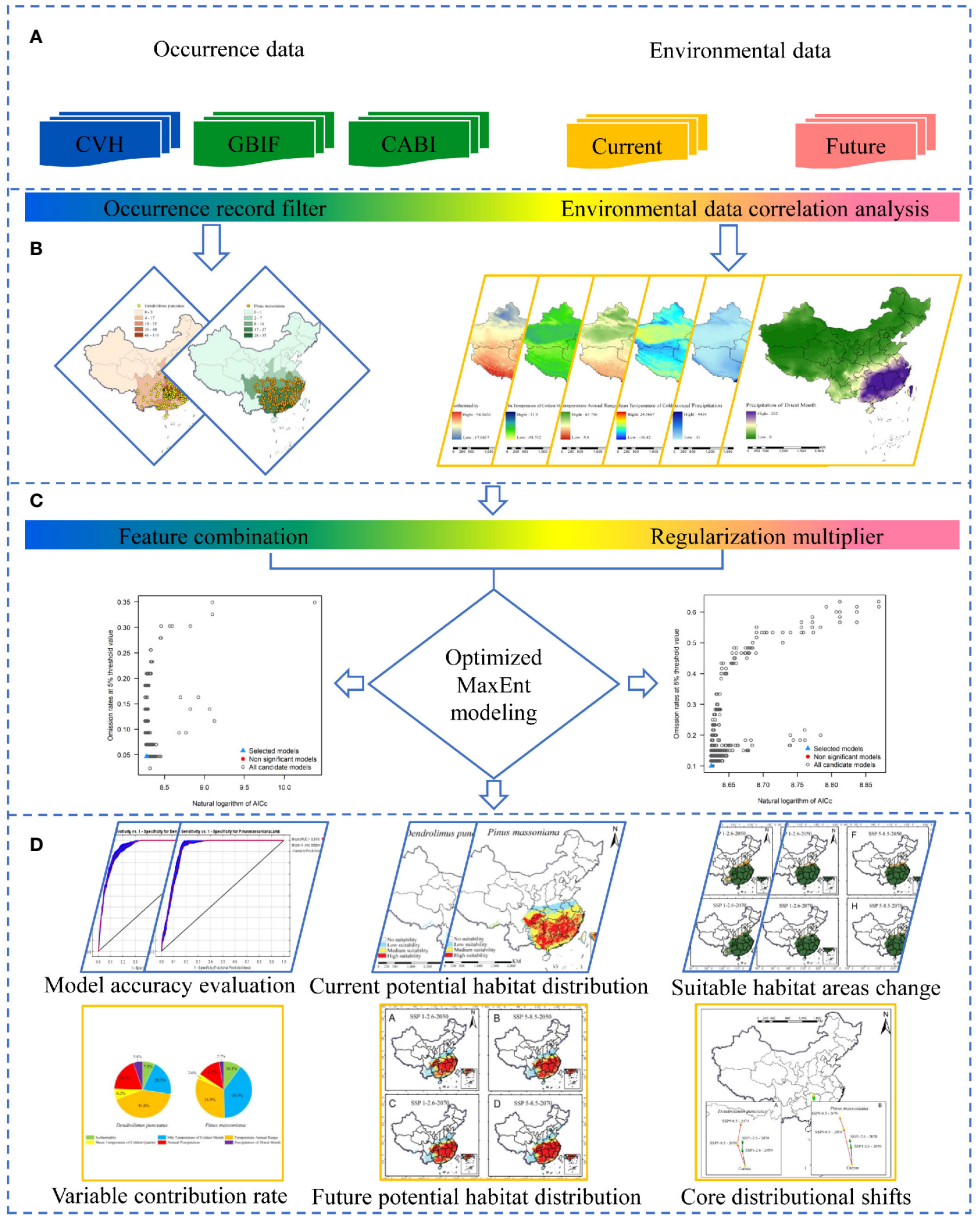
Research framework: **(A)** data collection, **(B)** data preprocessing, **(C)** optimization of the MaxEnt model, and **(D)** habitat suitability mapping and analysis of the results.

Aligned with our research objectives and the foundational principles of the MaxEnt model, we developed a framework to simulate the potential distribution of *D. punctatus* and *P. massoniana* and evaluate the spatiotemporal variations in their future suitable habitats under the influence of climate change. The framework is segmented into four key sections: (1) data collection; (2) data preprocessing; (3) optimization of the MaxEnt model; and (4) mapping of suitable habitats and analysis of the results (**Figure 3**). In the first section, we compiled occurrence records for *D. punctatus* and *P. massoniana*, along with current and future environmental variables that influence their distribution. The second section involved filtering the occurrence records to remove duplicates and samples that did not fit within the range of environmental variables. We also reduced redundancy in the environmental data by analyzing the contributions and correlations of environmental variables. In the third section, two critical parameters of the MaxEnt model were optimized to enhance the accuracy of the predictions. Finally, in the fourth section, using the processed data and the optimized MaxEnt model, we simulated the potential distribution areas of both species and predicted their future distributions. We then conducted an analysis and discussion of the prediction results and key environmental factors.

### 3.2 MaxEnt model

#### 3.2.1 Model description

The MaxEnt model was employed to predict the potential geographic distribution range of *D. punctatus* and *P. massoniana*. This model adopts the principle of maximum entropy, which is a statistical axiom asserting that the probability distribution with the highest entropy represents the best estimate of an unknown probability distribution when limited information is available (Phillips et al., 2006). This principle suggests that a system, in the absence of external constraints, will naturally gravitate toward a state of maximum entropy. Under specific conditions, the state of maximum entropy is most likely to resemble the system’s true state (Jaynes, 1957). Two types of data are essential when applying MaxEnt to species distribution modeling. The first type is the known geographical distribution of the species, represented by latitude and longitude data. The second type of data encompasses the environmental variables within the predictive spatial range. In deploying the maximum entropy principle for species distribution prediction, we utilize environmental variables and species presence data to establish constraints for the model. These constraints are based on the congruence of two mathematical expectations: one considers the mathematical expectation of each environmental variable under an unknown probability distribution; and the other focuses on the mathematical expectation of each environmental variable within the species presence data under a uniform distribution. The optimal prediction should adhere to these constraints while possessing the maximum information entropy. The MaxEnt model is essentially a constrained optimization algorithm. In this model, given an input *x* resulting in an output *y*, and for a specific training dataset and feature functions fi(*x*, *y*), where *i* = 1, 2,…, *n*, MaxEnt determines the optimal solution by solving a series of equations. These equations aim to maximize the information entropy of the overall system while fulfilling all known constraints. The MaxEnt equation-solving process is described as follows:


$$\text{ma}{\text{x}}_{\text{p}\in \text{c}}\text{H}\left(\text{P}\right)=\sum_{\text{x},\text{y}}\tilde{\text{P}}\left(\text{X}\right)\text{P}(\text{y}|\text{x})\text{logP}(\text{y}|\text{x}),$$

$$\text{s}.\text{t}{\text{E}}_{\text{P}}\left({\text{f}}_{\text{i}}\right)={\text{E}}_{\tilde{\text{P}}}\left({\text{f}}_{i}\right),\text{i}=1,2,3\ldots ,\text{n},$$

$$\sum_{\text{y}}\text{P}(\text{y}|\text{x})=1$$

where *H*(*P*) represents the conditional entropy; *P*(*y*|*x*) is the assumption of the conditional probability distribution; $\tilde{P}$ denotes the empirical distribution; and *E_p_* (*f_i_*) is the expected value of the feature function relative to the empirical distribution. The equation is solved using the Lagrangian multiplier method, which effectively converts the original constrained optimization problem into an unconstrained dual problem, thereby streamlining the optimization process within the framework of the MaxEnt model.

#### 3.2.2 Model optimization

Research indicates that using default parameters in MaxEnt modeling can lead to excessive complexity and the poor portability of the model (Morales et al., 2017; Wiltshire and Tanner, 2020). This phenomenon is closely associated with two critical parameters in the MaxEnt model: feature combination (FC) and the regularization multiplier (RM) (Akaike, 1974; Zhu and Qiao, 2016). We utilized the ‘Kuenma’ package in R version 3.6.3 to optimize the RM and feature categories in the MaxEnt model. During the modeling process, all occurrence records were randomly divided into a training (75%) and test (25%) set. We created 1160 candidate models, covering 40 different RM settings ranging from 0.1 to 4 (in increments of 0.1) and 29 different combinations of feature categories. The selection of candidate models was based on three criteria: (1) statistical significance; (2) an omission rate less than 5%; and (3) a model complexity (the minimum information criterion AICc value, delta.AICc) less than 2 (Cobos et al., 2019). We first filtered out statistically significant models and then retained those within the models that met the omission rate criterion (E< 5%). Finally, we selected the model that performed best in terms of significance, omission rate, and complexity. Reliable models typically have a delta AICc< 2, with delta AICc = 0 considered as the optimal model (Zhan et al., 2022). Based on the AICc values, we identified the optimal combination of FC and RM parameters (**Table 2**).

**Table 2:** Performance of the MaxEnt model under the optimal parameters.

Species	Type	RM	FC	delta AICc
*D. punctatus*	Default	1	LQPH	127.313
	Optimization	0.1	LP	0
*P. massoniana*	Default	1	LQPH	30.6034
	Optimization	0.1	QP	0

### 3.3 Model construction and validation

During construction of the model, 75% of the geographic distribution data was used for model training, and the remaining 25% was used for model validation. To enhance reliability of the results, we established 10 replicates and employed bootstrapping as the sampling method. The Logistic function was used as the output format, and the final results represent the average of the 10 replicates. We subsequently adopted the jackknife method to assess the relative importance of each climatic factor within the suitable areas for *D. punctatus* and *P. massoniana*, thus identifying the key limiting factors affecting the distribution of these two species (Phillips et al., 2006). The accuracy of the model was evaluated by determining the area under the curve (AUC) of the receiver operating characteristic (ROC) curve. The AUC value is not influenced by specific thresholds, hence is widely used to assess the accuracy of predictive models. The AUC ranges from 0 to 1 and is directly proportional to the accuracy of a model. In general, AUC values less than 0.7 indicate poor model performance; values between 0.7 and 0.8 denote moderate performance; values between 0.8 and 0.9 indicate good performance; and values greater than 0.9 suggest excellent performance. The closer the AUC value is to 1, the better the predictive performance of the model (Zhao et al., 2021).

### 3.4 Variations in the spatial pattern of the suitable distribution area

The MaxEnt model outputs the probability of species presence (p) in each grid cell in ASCII format, with values ranging from 0 to 1. We visualized the model’s output in ArcGIS and employed the reclassification tool to categorize suitability levels, calculating the corresponding area for each category. Based on the suitability index, the suitable areas were divided into four levels: unsuitable (p< 0.1), low suitability (0.1< p< 0.3), moderate suitability (0.3< p< 0.5), and high suitability (p > 0.5) (Yan et al., 2021).

We defined the suitable areas (assigned a value of 1) for *D. punctatus* and *P. massoniana* as spatial units with p > 0.1 and areas with p< 0.1 as unsuitable (assigned a value of 0) (Zhao et al., 2021). Based on this principle, we established potential distribution matrices for these species under current and future climate change scenarios, where 0 indicates absence and 1 indicates presence. This approach allowed us to analyze the spatial pattern changes in suitable distribution areas under future climate scenarios. Changes in areas for 2050 and 2070 were calculated based on current and projected distributions for 2050. Moreover, we defined four types of changes: newly suitable areas (matrix value changing from 0 to 1); lost suitable areas (from 1 to 0); retained suitable areas (1 remaining constant); and consistently unsuitable areas (0 remaining constant).

To further analyze the dynamics of species distribution, we simplified the distribution of *D. punctatus* and *P. massoniana* to a single centroid point and created a vector file to describe the magnitude and direction of changes in the suitable areas over time. By tracking the centroid changes under different climate scenarios, we could explore the dynamics of species distribution. Furthermore, we calculated the cosine similarity of the changes in the distribution centroids of *D. punctatus* and *P. massoniana* under the same scenarios to determine whether the migration directions of these two species were similar.

## 4 Results

### 4.1 Model accuracy evaluation

The MaxEnt model employs the ROC curve to assess the accuracy of the analytical results. The ROC curve is a tool for evaluating the performance of classification models, plotting the false positive rate on the x-axis against the true positive rate on the y-axis. The area between the curve and the x-axis, denoted as the AUC, is used to quantify the overall performance of the model. The average AUC values (obtained through 10 replicate runs of the distribution models) for *D. punctatus* and *P. massoniana* were 0.931 and 0.923, respectively, indicating that both models exhibit excellent performance, and their predictive accuracy is reliable. Furthermore, the high AUC values indicate the efficiency of the models in differentiating between suitable and unsuitable areas, thereby providing robust scientific support for our research.

### 4.2 Environmental variable analysis

**Figure 4:**
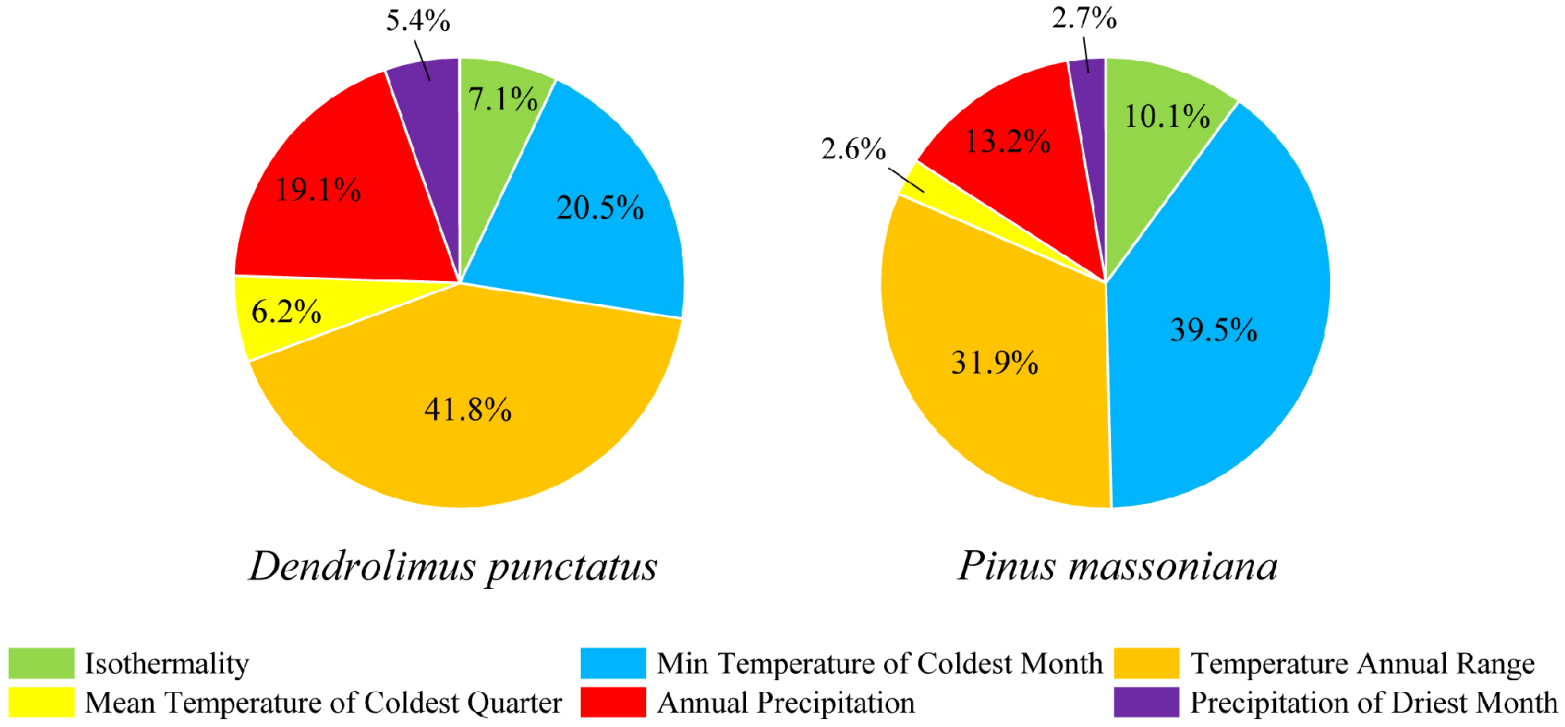
Percentage contributions of environmental variables used in the final MaxEnt model. On the left is *D. punctatus* and on the right is *P. massoniana*.

We determined the key environmental variables influencing the species distribution by analyzing the percentage contributions of various environmental variables within the predictive model. Six environmental variables were selected for analysis (**Figure 4**). Among these variables, the minimum temperature of the coldest month (bio06), annual temperature range (bio07), and annual precipitation (bio12) collectively contributed to 81.4% and 84.6% of the overall impact of *D. punctatus* and *P. massoniana*, respectively. This highlights the significance of these variables as the three main environmental driving factors in the distribution of *D. punctatus* and *P. massoniana*. The remaining contribution, accounting for less than 20%, was jointly provided by isothermality (bio03), the mean temperature of the coldest quarter (bio11), and the precipitation of the driest month (bio14).

### 4.3 Current distribution of *D. punctatus* and *P. massoniana*

**Figure 5:**
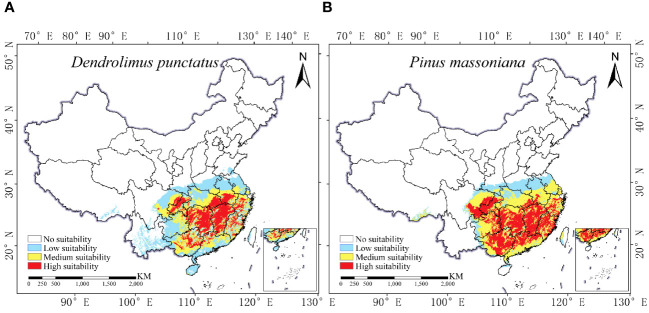
Current distribution of *D. punctatus* and *P. massoniana* in China. **(A)** *D. punctatus*; **(B)** *P. massoniana*.

**Figure 5** reveals that *D. punctatus* and *P. massoniana* are primarily distributed in regions south of 35°N latitude in China, consistent with existing literature. The distribution areas of both species exhibit significant similarity, with their northern boundaries aligning with the Qinling Mountains and the Huai River, and their southern boundaries extending to Hainan Island. The total area of their distribution is approximately 193.98 × 10^4^ km^2^ for *D. punctatus* and 191.43 × 10^4^ km^2^ for *P. massoniana*, respectively. The high-suitability areas for *D. punctatus* are mainly concentrated in Hunan and Jiangxi Provinces, followed by Chongqing, Hubei, Henan, Anhui, Jiangsu, Zhejiang, Fujian, and Guangdong Provinces and Guangxi Zhuang Autonomous Region. In contrast, the high-suitability areas for *P. massoniana* are more extensive, stretching eastward to the coastal regions of China, covering the central part of Guangdong Province and the southwestern region of Guangxi Zhuang Autonomous Region, as well as the eastern part of Guizhou Province.

### 4.4 Projected distribution of *D. punctatus* and *P. massoniana* under future climate scenarios

**Figure 6:**
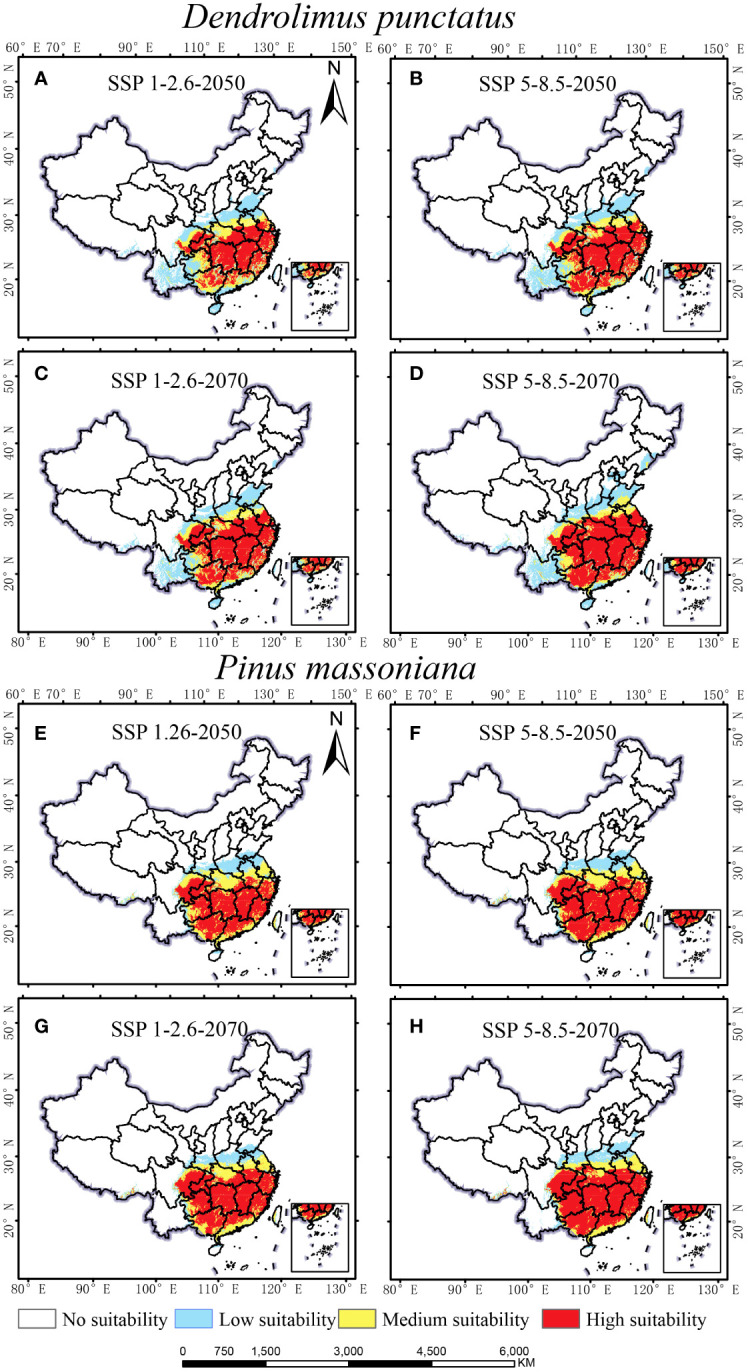
Suitable habitat maps for *D. punctatus* and *P. massoniana* under two different future scenarios. **(A)** *D. punctatus* SSP1-2.6-2050; **(B)** *D. punctatus* SSP5-8.5-2050; **(C)** *D. punctatus* SSP1-2.6-2070; **(D)** *D. punctatus* SSP5-8.5-2070; **(E)** *P. massoniana* SSP1-2.6-2050; **(F)** *P. massoniana* SSP5-8.5-2050; **(G)** *P. massoniana* SSP1-2.6-2070; **(H)** *P. massoniana* SSP5-8.5-2070.

The impact of climate change on the distribution of *D. punctatus* and *P. massoniana* may increase the risk of pest outbreaks. **Figure 6** presents the projected distribution of suitable habitats for these species under different future climate scenarios. Predictions indicate that the distribution range of both species will continue to expand in the coming decades. Particularly under the future SSP1-2.6 scenario (**Figures 6A, C**), the suitable habitat of *D. punctatus* is expected to expand north-eastward to northern Shandong, with the suitable habitat area reaching 224.68 × 10^4^ km^2^ in 2050 and 235.75 × 10^4^ km^2^ in 2070. Under the SSP5-8.5 scenario (**Figures 6B, D**), the north-eastward expansion trend of *D. punctatus* is even more pronounced, with the suitable habitat area estimated at 240.02 × 10^4^ km^2^ in 2050 and 267.78 × 10^4^ km^2^ in 2070. Moreover, under both climate scenarios, the high-suitability area for *D. punctatus* markedly increases, suggesting that future climatic conditions may be more conducive to the survival of this species and could increase the risk of outbreaks. In contrast, the northward expansion of *P. massoniana* is slower (**Figures 6E–H**), but the high-suitability area for this species continues to increase under both climate scenarios, indicating that southern regions of China will become more suitable for the growth of *P. massoniana*. However, the suitable habitat of *D. punctatus* almost entirely overlaps with the suitable area for *P. massoniana*, suggesting that *P. massoniana* may continue to suffer from the pest in the future.

## 5 Discussion

### 5.1 Impact of environmental variables on the distribution of *D. punctatus* and *P. massoniana*

Environmental variables are widely acknowledged as key factors affecting species distribution patterns. These variables impact the growth, development, and interspecies interactions of species (Raza et al., 2015). Future climate change is anticipated to markedly affect the distribution of *D. punctatus* and *P. massoniana*. In our study, the primary environmental variables influencing the distribution of *D. punctatus* and *P. massoniana* are identified as the minimum temperature of the coldest month (bio06), annual temperature range (bio07), and annual precipitation (bio12). Among these factors, those related to temperature contribute more significantly, indicating a higher sensitivity of *D. punctatus* and *P. massoniana* to temperature variations. Concurrently, annual precipitation is also instrumental in the spatial distribution modeling of these two species. This finding aligns with conclusions drawn from physiological and ecological studies of *D. punctatus* and *P. massoniana* (Zeng et al., 2010; Lei and Wang, 2024). The development of *D. punctatus* requires a certain amount of accumulated temperature. In the distribution areas of this species, from north to south, the number of generations completed per year increases with the rise in average annual temperature. Precipitation influences the occurrence of pest outbreaks by altering air humidity and through the washing effect on the larvae of *D. punctatus*. *P. massoniana*, preferring a light-abundant and deep-rooted environment, thrives in warm and moist climates and is typically found in regions with distinct seasons and concurrent periods of heat and rainfall (Fei et al., 2014; Wang et al., 2016; Yan et al., 2019). The distribution area of *D. punctatus* lies within China’s subtropical monsoon and tropical monsoon climate zones, characterized by hot summers, mild winters, minimum average temperatures above 0°C in the coldest months, and annual rainfall ranging from 1000 to 2000 mm. This region predominantly features *P. massoniana* as its representative vegetation. Suitable climatic conditions and extensive *P. massoniana* forests provide favorable conditions for outbreaks of *D. punctatus*. In addition to temperature and precipitation, environmental factors such as elevation, solar radiation intensity, predation competition, and extent of vegetation cover significantly influence the distribution of insects and plants (Li et al., 2023). For example, during the hatching period of the *D. punctatus*, larvae often disperse with the wind, with their direction of spread being influenced by the wind direction. The dispersal process is affected by wind force, wind speed, and topography (Chen, 1990). Soil pH values can impact the distribution of *P. massoniana*, a species that prefers acidic soils and is intolerant to saline conditions. Appropriate climatic and soil environments are essential for the formation of *P. massoniana* forests. Changes in *P. massoniana* forests can also affect the distribution patterns of the *D. punctatus*. However, integrating all influencing factors into a single model to simulate the potential distribution of species is a challenging task. Moreover, introducing too many variables may lead to increased multicollinearity issues, diminishing the impact of key variables. Nonetheless, the predictions of future suitable habitat migration changes in this study are consistent with the growth habits of *D. punctatus* and *P. massoniana*, and thus our results are a valuable reference for potential suitability forecasts of these two species under the context of climate change.

### 5.2 Changes in the distribution areas of *D. punctatus* and *P. massoniana* under future climate scenarios

**Figure 7:**
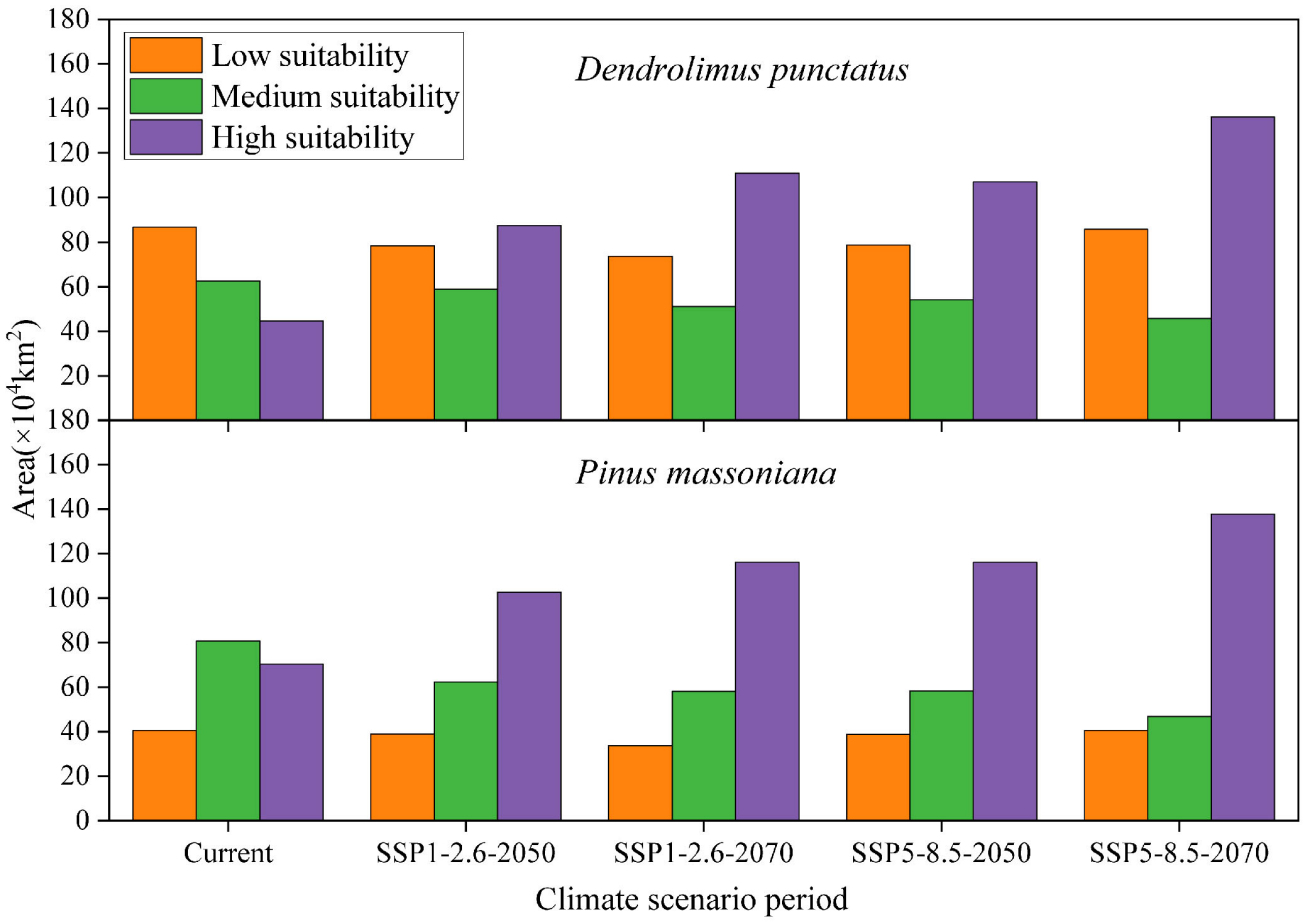
Suitable habitat areas of *D. punctatus* and *P. massoniana* under different climate scenarios.

**Figure 8:**
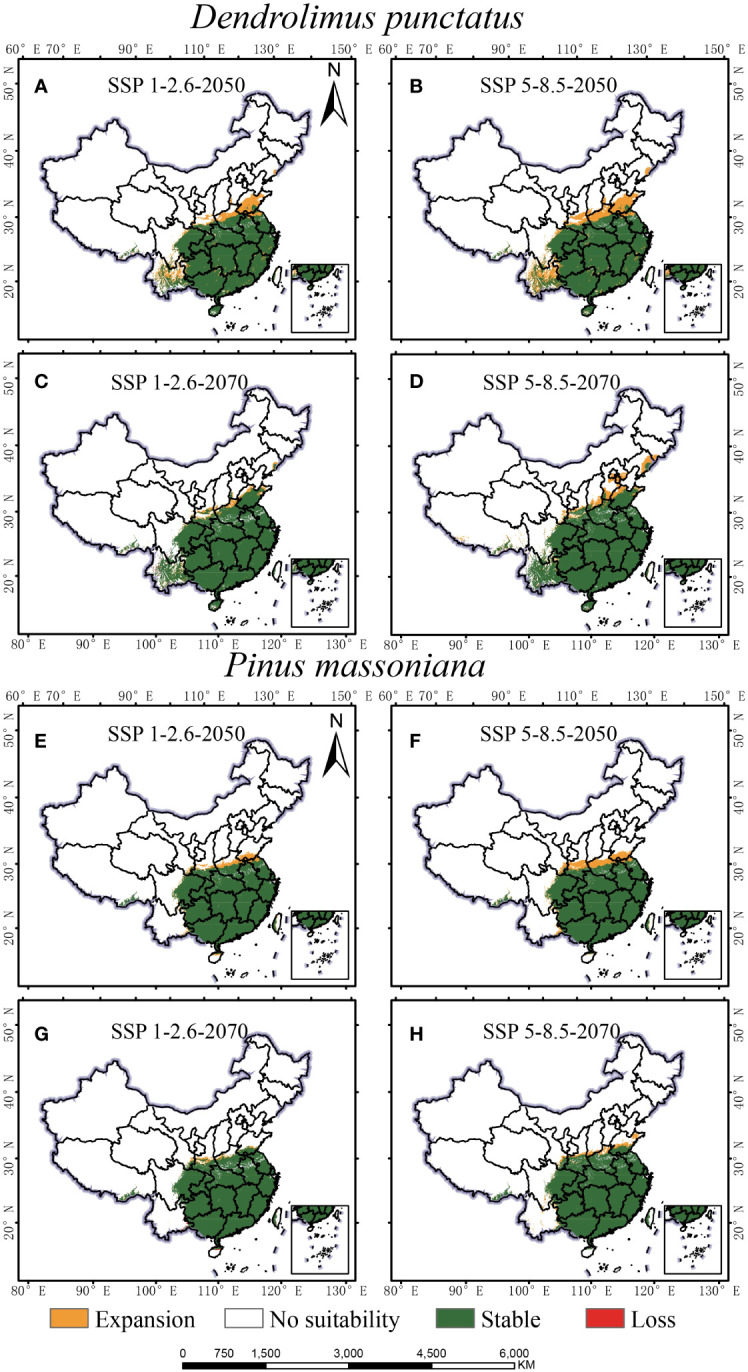
Geographic distribution changes of *D. punctatus* and *P. massoniana* under different future climate scenarios. **(A)** *D. punctatus* SSP1-2.6-2050; **(B)** *D. punctatus* SSP5-8.5-2050; **(C)** *D. punctatus* SSP1-2.6-2070; **(D)** *D. punctatus* SSP5-8.5-2070; **(E)** *P. massoniana* SSP1-2.6-2050; **(F)** *P. massoniana* SSP5-8.5-2050; **(G)** *P. massoniana* SSP1-2.6-2070; **(H)** *P. massoniana* SSP5-8.5-2070.

Under all future climate scenarios, the total distribution areas of *D. punctatus* and *P. massoniana* are projected to increase to varying degrees compared to the present, generally showing a trend of northward expansion (**Figures 7**, **8**). By 2070, under the SSP5-8.5 scenario, predictions indicate that the suitable habitat areas for these two species will reach their maximum. Numerous studies have highlighted that climate change may cause significant shifts in species distribution patterns. Under the SSP1-2.6 scenario, by 2050, the new areas for *D. punctatus* are expected to include central Shandong, Henan, parts of Yunnan, and some areas of Liaoning (**Figure 8A**). By 2070, although the suitable area for *D. punctatus* continues to expand northward, the increase is relatively modest, and the suitable areas in the Yunnan region will have decreased slightly compared to 2050 (**Figure 8C**). Under the SSP5-8.5 scenario, the changes in suitable habitat for *D. punctatus* are more significant, expanding overall toward the northeastern region, covering areas from southern Gansu through Shaanxi, Shanxi, Hebei, Beijing, and Tianjin, and extending to Liaoning and parts of Jilin (**Figures 8B, D**). In future periods, the expansion area of the suitable distribution of *D. punctatus* generally exceeds its contraction area, indicating that future climatic conditions will be more favorable for the survival of this species. In contrast, the changes in suitable habitat for *P. massoniana* are relatively small but also exhibit a trend of northward expansion (**Figure 8**). In both future climate scenarios, the changes in suitable habitat for *P. massoniana* are broadly consistent, showing a trend of northward expansion by 2050 (**Figures 8E, F**), with the expansion area mainly including Henan and Shandong, as well as central Shanxi and parts of Gansu in the SSP5-8.5 scenario. By 2070, the suitable habitat for *P. massoniana* remains relatively stable under both climate scenarios, with only a small part of the area experiencing expansion (**Figures 8G, H**). In these future climate scenarios, the stable areas of suitable habitat for *D. punctatus* and *P. massoniana* are essentially consistent, implying that *P. massoniana* will continue to face pest risks in the future.

### 5.3 Changes in the habitat centroids of *D. p unctatus* and *P. massoniana* under future climate scenarios

**Figure 9:**
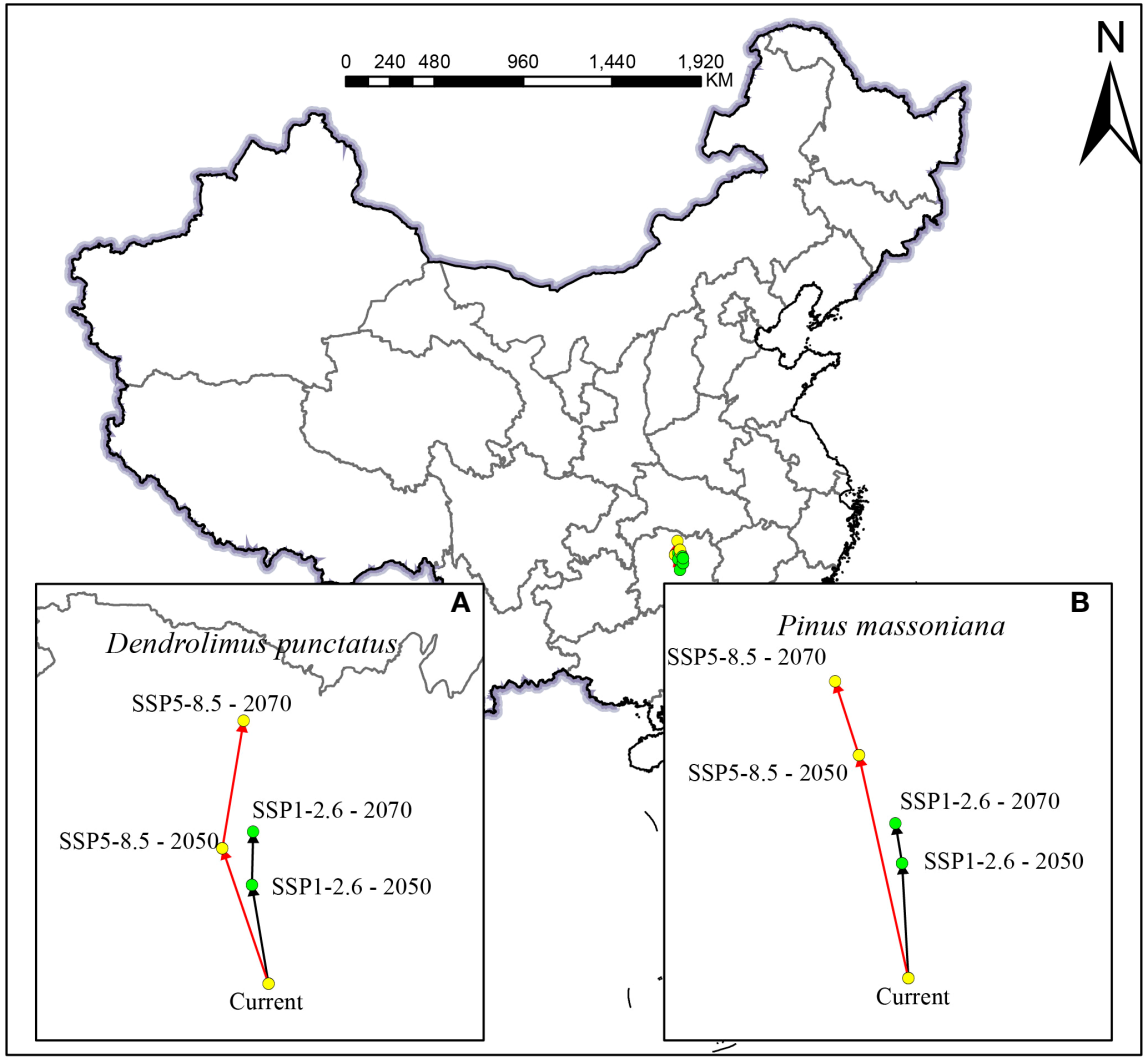
Spatial changes of the geometric centroids of suitable habitat areas by 2050 and 2070 under two different climate change scenarios: **(A)** *D. punctatus*; **(B)** *P. massoniana*.

Climate change is likely to cause significant changes in species distribution patterns, prompting their northward migration (Chen et al., 2022). Long-term climate observations indicate that, with the global warming trend, China’s annual average temperature is expected to rise by approximately 2.6°C, particularly under the high greenhouse gas emission scenario SSP5-8.5, where the temperature increase will be more pronounced. Concurrently, the annual average precipitation is projected to increase by 5.2%, particularly in North China and the Northwest region (Fu and Jiang, 2011). These changes are anticipated to further promote the northward migration of many species. Under current climatic conditions, the centroids of suitable areas for *D. punctatus* and *P. massoniana* are situated in Hunan Province (**Figure 9**). Model predictions for the SSP1-2.6 scenario suggest that from the present to 2050, the centroid of the suitable habitat for *D. punctatus* will move 59.37 km to the northwest, and from 2050 to 2070, it will further move 31.52 km to the northeast., respectively, resulting in a total northward shift of 90.48 km. Under the SSP5-8.5 scenario, from the present to 2050, the centroid of the suitable habitat for *D. punctatus* is projected to move 84.85 km northwest, and from 2050 to 2070, it is expected to shift 76.54 km northeast, totaling a northward movement of 156.43 km. The suitable habitat centroid of *P. massoniana* also shifts northward, although the extent of migration is smaller, moving northward by 37.20 km and 73.09 km under the two future climate scenarios by 2070. This may be associated with the ecological characteristics of *P. massoniana*, whose needle-like leaves effectively prevent water evaporation, thus reducing its sensitivity to temperature changes. These results indicate that the suitable areas for *D. punctatus* and *P. massoniana* are expected to expand and migrate toward higher latitude regions with increasing temperature and precipitation, aligning with their preference for warm and humid environments. Global warming facilitates the spread of insects limited by low temperatures to higher latitude areas. Future trends of surface warming in China intensifying toward higher latitudes and the Tibetan Plateau, coupled with increased winter precipitation in the north, provide favorable conditions for the northward expansion of *D. punctatus*. Under different future climate scenarios, the movement directions and ranges of *D. punctatus* and *P. massoniana* show similarities, indicating that *P. massoniana* may face greater pest risks in the future.

Therefore, in the context of climate change, it is vital to effectively manage *D. punctatus* in various suitable habitats and to protect *P. massoniana* resources. Strengthening pest detection and adopting more proactive management practices are essential. Enhanced pest control measures in high-suitability areas for *D. punctatus* are critical, whereas in low-suitability areas, considering ecosystem conservation, reducing the use of chemical agents in favor of natural control methods is recommended. For *P. massoniana*, trunk injection technology can potentially be employed as an effective method to control pest infestations.

### 5.4 Similarity in centroid shift changes between *D. punctatus* and *P. massoniana*

In this study, we employed the statistical method of cosine similarity to assess the similarity in the direction of centroid migration for *D. punctatus* and *P. massoniana* under different future climate scenarios and at two time points. Cosine similarity measures the similarity between two vectors by calculating the cosine of the angle between them. A cosine similarity value of 1 indicates that the two vectors are in the same direction, while a value of −1 indicates they are in opposite directions. Under the scenarios SSP1-2.6 and SSP5-8.5, from the present to 2050, the shift in distribution centroids for *D. punctatus* and *P. massoniana* showed a high degree of similarity, with a similarity index of 0.99. This indicates that the migration directions of the two species are almost identical. Furthermore, under the SSP5-8.5 scenario by 2050, their distribution centroids of *D. punctatus* and *P. massoniana* are closest, at a mere distance of 25.18 km. These findings reveal a critical insight: the period from the present to 2050 could be a high-risk phase for *P. massoniana* forests due to *D. punctatus* infestation, making this interval crucial for the control and management of *D. punctatus* in pine forests. This discovery holds significant implications for formulating future pest control strategies and management measures. The similarity in the shift directions of the distribution centroids for the two species between 2050 and 2070 under both scenarios also remains high, at 0.97 and 0.85, respectively. However, if proactive control measures against *D. punctatus* are implemented in the first phase, pest issues may be mitigated in the latter half of the century.

In this study, we focused on analyzing the potential distribution changes of *D. punctatus* and *P. massoniana* under future climate change scenarios. However, the model does not encompass all key factors that could influence the distribution of these two species, such as geographical barriers, natural enemies, human activities, and land use. Despite this, currently, no comprehensive model exists that can integrate all these factors for species distribution prediction (Guan et al., 2022). The future suitable habitats predicted in this study for the two species align with their growth habits, providing a valuable reference for understanding the habitat changes and migration directions of *D. punctatus* and *P. massoniana* in the context of climate change. Future research should consider a broader range of influencing factors to develop more comprehensive species distribution prediction models, which would enable more accurate predictions of the distribution of these two species.

### 5.5 Management and control recommendations

The impact of climate change on ecosystems has become increasingly evident, leading to the northward spread of forest pests including *Monochamus alternatus* in China and the general trend of the potato pest *Schrankia costaestrigalis* moving toward the northeast and higher latitudes (Xu et al., 2020; Xian et al., 2023). In our study, the increases in two primary factors of climate change—temperature and precipitation—are key in influencing the spread of *D. punctatus*. As global climate change intensifies, global warming has significantly accelerated the northward expansion of *D. punctatus*. This phenomenon is concerning in that, in the not-too-distant future, *D. punctatus* may continue to migrate northward in search of new habitats, posing a greater threat to tree species in the northern regions. In addition, our research found that *D. punctatus* and *P. massoniana* (its primary host) share similar geographic distributions and migration trends. This finding suggests that in the future, with the further intensification of climate change, *P. massoniana* may face more severe pest risks. Therefore, our study underscores the importance of monitoring and managing these pest migration trends to mitigate their potential impact on ecosystems.

This study outlines three pivotal strategies for managing *D. punctatus* and conserving *P. massoniana*: (1) Given the projected suitable habitats in the current climate change scenario, enhancing surveillance and early warning systems for *D. punctatus* is critical. This encompasses promptly identifying and managing pest outbreaks to ensure swift and effective containment; (2) Considering the anticipated distribution patterns of *D. punctatus*, particularly in newly affected regions in northern China, reinforcing preventative measures is imperative. Forestry practices, in establishment and regeneration, should aim to modify habitats to create conditions less conducive to the proliferation of *D. punctatus*. This holistic approach is vital for curbing the spread of *D. punctatus* and mitigating its detrimental effects on ecosystems; (3) In *P. massoniana* forests already experiencing *D. punctatus* infestation, introducing broadleaf species and creating mixed coniferous-broadleaf forests are recommended. This strategy curbs the likelihood of extensive, high-density *D. punctatus* outbreaks and bolsters the overall resilience and stability of forest ecosystems. Additionally, utilizing the natural predators of *D. punctatus* (e.g., *Trichogramma*) for biological control is advisable. Cultivating and periodically releasing Trichogrammatid in suitable regions could serve as an effective natural control mechanism, preventing widespread *D. punctatus* infestations and thus safeguarding *P. massoniana* resources.

## 6 Conclusion

This study analyzed the distribution patterns of *D. punctatus* and its host *P. massoniana* based on occurrence records and current and future climate data. The MaxEnt model, parameterized through optimization, was employed to predict the distribution of both species under current and future conditions. The results indicate that under current climate conditions, *D. punctatus* and *P. massoniana* are primarily distributed in the region south of China’s Qinling–Huaihe line. The main environmental variables influencing the distribution of both species are related to temperature and precipitation, including the lowest temperature of the coldest month, the annual temperature range, and annual precipitation. Under future climate conditions, the suitable habitat area for *D. punctatus* is expected to increase and shift toward higher latitudes. In the SSP5-8.5 climate scenario, characterized by increased greenhouse gas emissions and intensified global warming, *D. punctatus* is projected to expand further toward higher latitudes. The similarity in the migration direction between the two species is remarkably high, reaching 0.99 in the SSP5-8.5 scenario by 2050. Meanwhile, the distance between the distribution centroids of *D. punctatus* and *P. massoniana* is only 25.18 km during this period, signifying a critical phase for preventing and managing *D. punctatus* infestations in *P. massoniana* forests. Although the future suitable habitat and migration direction of *P. massoniana* are highly similar to those of *D. punctatus*, the changes are relatively slow, indicating that *P. massoniana* will continue to be affected by *D. punctatus* infestations in the long term. This study on the distribution of *D. punctatus* and *P. massoniana* provides valuable theoretical insights for the future prevention of *D. punctatus* infestations and the conservation of *P. massoniana* resources.

## Data availability statement

The original contributions presented in the study are included in the article/supplementary material. Further inquiries can be directed to the corresponding authors.

## Author contributions

YW: Conceptualization, Data curation, Formal analysis, Investigation, Methodology, Project administration, Software, Visualization, Writing – original draft, Writing – review & editing. ZY: Project administration, Writing – review & editing. GM: Writing – review & editing, Conceptualization, Funding acquisition, Methodology, Supervision. XZ: Project administration, Writing – review & editing. CY: Writing – review & editing, Project administration. YC: Funding acquisition, Methodology, Supervision, Validation, Writing – review & editing.
